# Therapeutic Effects of IL-1RA, M2 Cells, and Their Synergistic Impact on a Mouse Model of Rheumatoid Arthritis

**DOI:** 10.34172/apb.2024.037

**Published:** 2024-03-11

**Authors:** Mohammad Sadegh Hashemzadeh, Hadi Esmaeili Gouvarchin Ghaleh, Mozafar Mohammadi, Yaser Yousefpoor, Ehsan Rezaei, Gholamhossein Alishiri

**Affiliations:** ^1^Nanobiotechnology Research Center, Baqiyatallah University of Medical Sciences, Tehran, Iran.; ^2^Applied Virology Research Center, Baqiyatallah University of Medical Sciences, Tehran, Iran.; ^3^Applied Biotechnology Research Center, Baqiyatallah University of Medical Sciences, Tehran, Iran.; ^4^Department of Medical Biotechnology, School of Paramedical Sciences, Torbat Heydariyeh University of Medical Sciences, Iran.; ^5^Molecular Biology Research Center, Baqiyatallah University of Medical Sciences, Tehran, Iran.; ^6^Chemical Injuries Research Center, Baqiyatallah University of Medical Sciences, Tehran, Iran.

**Keywords:** Rheumatoid arthritis, IL-1RA, Anti-inflammatory macrophages, Synergistic effect, Novel therapeutic approach, Combinatory therapy

## Abstract

**Purpose:**

Rheumatoid arthritis (RA) is a type of autoimmune disease that results in chronic inflammation of the joint synovial tissue, leading to joint damage and significant disability. Despite ongoing research, the exact cause of RA remains unclear, and current treatments have limitations. This study explores the potential of utilizing interleukin-1 receptor antagonist (IL-1RA) and anti-inflammatory macrophages polarized in the vicinity of the supernatant from allogeneic mesenchymal stem cells (MSCs) as a novel therapeutic approach for RA.

**Methods:**

An expression cassette containing the *IL-1RA* gene was constructed and expressed in *E. coli* BL21. The resulting protein was purified and stabilized for use in in vivo experiments. Bone marrow MSCs were isolated and used to produce anti-inflammatory M2 macrophages from the isolated peripheral blood monocytes. The macrophages were then used to treat mice with RA induced by collagen type II.

**Results:**

The combination of IL-1RA and M2 macrophages improved clinical and histopathological symptoms of the disease, reduced levels of inflammatory factors, and modulated the immune system in the treated mouse groups. The results showed that this combinatory therapy had a synergistic effect for RA treatment.

**Conclusion:**

The simultaneous use of IL-1RA and M2 cells could be a promising approach for the treatment of RA. This combinatory therapy has the potential to improve the disease and decrease the severity of inflammation in patients with RA.

## Introduction

 Rheumatoid arthritis (RA) is a systemic autoimmune disease with chronic inflammation in the joint synovial tissue which leads to the destruction of several joints and finally severe disability. It is one of the most common inflammatory joint diseases which involve 1% of the population.^1^ The exact cause of RA is unknown. In the development of this disease, cellular and humoral immunity both play the key role, and the influence of heredity, infection, anxiety and gender has been observed in many patients. Some of self-antigens or foreign antigens with same antigenic structure with self-antigens can initiate the autoimmune responses of TCD4 + lymphocytes and subsequently cause the release of inflammatory cytokines such as, interleukin-1 (IL-1), IL-6, tumor necrosis factor (TNF)-α and reduction of anti-inflammatory cytokines such as IL-10.^2^ Activated macrophages release degrading enzymes such as matrix metalloproteinase which causes joint damage and release of cartilage oligomeric matrix protein.^3^ Some of produced cytokines by activation of B cells trigger generating the anti-nuclear autoimmune antibodies.^4^

 Currently, the effect of cytokines in the pathogenesis of this disease has been observed in animal models and also in humans.^5^ One of the most important cytokines that play a role in causing inflammation is interleukin-1 (especially β). On the other hand, the cytokine that limits the IL-1 inflammatory effects is the antagonist of interleukin-1 receptor (IL-1RA). This cytokine is synthesized by many cells, especially monocytes, macrophages, fibroblasts, and also hepatocytes (as a liver acute phase protein). The secreted form of IL-1RA that has 152 amino acids, due to its similarity to IL-1 competitively binds to the IL-1 receptor.^6^ Usually, in people with RA, both IL-1 and IL-1RA levels are high but the level of IL-1RA in joint fluid or synovial fluid is not enough to inhibit the inflammatory effects of locally produced IL-1.^7^ It is expected that increasing the level of IL-1RA can reduce the negative effects of IL-1 include destruction of cartilage, bone loss, etc, resulting in improvement of the disease and decrease the severity of inflammation in people with RA.

 Despite the advances that have occurred in the treatment of RA, many patients still do not respond well to common treatments.^8^ Some of current treatments of RA are include glucocorticoids, conventional disease-modifying anti rheumatic drugs (cDMARDs), nonsteroidal anti-inflammatory drugs, and biologic agents, among which cDMARDs has long been the main treatment. Novel drug candidates have been specifically aimed at and created, including various cell types like B cells and fibroblast-like synoviocytes, as well as cytokines such as TNF-α, IL-1,^9^ IL-6, and granulocyte-macrophage colony-stimulating factor. Additionally, signaling pathways like Janus kinase family (JAK) and IL-1-receptor-associated signaling pathways have been targeted in the development process.^10^ By the progress of knowledge on the immunopathogenesis of RA, the cell therapy by mesenchymal stem cells (MSCs) has attracted increasing attention. The MSCs can prevent the functions of effector T cells (Teff) and B cells and raise the capacity of regulatory T cells (Tregs).^11^ The mouse MSCs significantly debilitated RA in collagen-induced arthritis (CIA) model, which is due to significantly lower inflammation and erosion score.^12,13^ An explanation for why activated MSCs can reduce inflammation is by inhibiting the functioning of M1 macrophages and promoting the activation of M2 macrophages. This is achieved by increasing the expression of IL-10 and indoleamine 2,3-dioxygenase.^12-14^ The problem was identified with the isolated exosomes derived from MSCs, which have demonstrated a noteworthy ability to reduce the presence of the M1 macrophage marker inducible nitric oxide synthase and encourage the M2 polarization process.^15,16^ In previous studies, the existence of a close relationship between macrophages and changes in the state of the inflammation has been observed.^14^ M1 cells produce inflammatory cytokines such as IL-1, IL-6, TNF-α, etc. and cause inflammation and tissue damage. On the other hand, M2 cells produce anti-inflammatory cytokines such as IL-10, TGF-β, etc, regulate the homeostasis of different tissues, and repair damaged tissues by producing substances such as VEGF.^3,17^

 In this research, we tried to investigate the role of expressed recombinant IL-1RA, together with anti-inflammatory M2 macrophages (produced in the vicinity of the supernatant from allogeneic MSCs), in the inhibition of RA in a mouse model of the disease. With success in improving RA disease, this combinatory method can be used in the clinical phase in the future.

## Methods

###  Design and synthesis of recombinant IL-1RA gene

 For designing the recombinant *IL-1RA* gene, its sequence was obtained from NCBI with GenBank accession no. M63101. Codon optimization for expression in the *E. coli* BL21 host was performed by NOVO Prolab and Genescript. The IL-1RA construct was synthesized in pET-28a^( + )^ expression vector. The *E. coli* DH5α and *E. coli* BL21 (T7 express) hosts were transformed by pET-28a^( + )^ vector. Some colonies were selected on LB agar with kanamycin (50 μg/mL) for plasmid extraction. After that the presence of recombinant plasmid containing *IL-1RA* gene was assessed in the hosts by PCR and sequencing (by Macrogen Company).

###  Expression and purification

 The IL-1RA gene, presented in the pET28a( + ) plasmid, was expressed in *E. coli* BL21 bacteria. To begin, a single favorable clone was selected and had grown in 100 mL LB Broth medium with 100 µg/mL kanamycin at 37 °C and 150 rpm for 16 hours. Afterward, the solution was diluted 50 times with new media and placed at 37 °C under continuous agitation at 150 rpm. In optical density^18^ of 0.6 at 600 nm, expression was induced by 1 mM IPTG and incubated at 37 °C and 150 rpm for 6 hours. The whole medium was centrifuged at 8000 rpm for 5 minutes. The harvested cells were suspended in 5 mL binding buffer (50 mM NaH2PO4, 300 mM NaCl and 10 mM imidazole) and sonicated at 75 W (15 seconds working and 30 seconds resting for a 4 minutes pulse and then cooled in an ice-water bath) by sonicator (Hielscher UIS250V). Driven cell extract was centrifuged at 14 000 rpm, 4 °C for 20 minutes.

 Soluble recombinant protein found in supernatant solution was used to purification using a nickel-nitrilotriacetic acid affinity column. Before starting the purification process, the column was prepared by using a binding buffer containing 20 mM imidazole at a pH of 8. The supernatant solution containing the protein was then added to the column, followed by washing with a washing buffer composed of 20 mM imidazole at a pH of 8.The purified recombinant protein was subsequently collected by elution using 250 mM imidazole. Finally, the column was washed again, this time using a 20 mM MES buffer with a pH of 5.5. The purified recombinant protein was evaluated using 12 % sodium dodecyl sulfate–polyacrylamide gel electrophoresis (SDS-PAGE). Quantifying recombinant protein versus bovine serum albumin standards was performed by Bradford method.^19,20^

###  Western blot analysis

 To validation of purified IL-1RA protein, western blot analysis was done. The IL-1RA protein was isolated through a 12 % SDS-PAGE and subsequently transferred onto a nitrocellulose membrane obtained from Sigma, USA. During the transfer process, a transfer buffer consisting of 39 mM glycine, 48 mM Tris base, 0.037 % SDS, and 20 % methanol was utilized. After blocking with 5 % skimmed milk, membrane incubation with 1:2000 diluted horseradish peroxidase (HRP)-conjugated Anti His_6_-Tag antibody (Sigma, Berlin, Germany) in PBS-T (PBS + 0.05 % Tween 20) at 4 °C for overnight was done. DAB (3,3′-Diaminobenzidine) substrate was applied to detection of the purified IL-1RA protein.

###  Isolation and culture of mouse bone marrow MSCs

 BALB/c mice (6-8 weeks) were used to isolation of bone marrow. The mice were euthanized using cervical dislocation. Subsequently, the tibia and femur bones were cut at their ends to facilitate bone exposure. The marrow separated of the cut end of the bone flushed to extract the cells by 5-mL syringe containing complete media and collected in a 15-mL tube. The suspension underwent centrifugation at 12 000 rpm for 10 minutes. Subsequently, it was resuspended in 2 mL of Dulbecco’s modified Eagle’s medium (DMEM) high glucose (manufactured by Gibco, USA) along with 10% fetal bovine serum (FBS; manufactured by Gibco, USA) and 100 U/mL penicillin-streptomycin (manufactured by Gibco, USA). This mixture was then cultured in a 25-cm^2^ culture flask and incubated at a temperature of 37 °C with a 5% CO2 atmosphere. After 72 hours, non-adherent cells were eliminated, and the flask was rinsed with phosphate-buffered saline (PBS; manufactured by Gibco, USA). Regular medium changes were executed every 2-3 days, and once the cells reached 80% confluency, they were detached using trypsin-EDTA (manufactured by Gibco, USA) and transferred to new flasks.

###  Monocyte isolation 

 The blood that was collected from the mice under deep anesthesia was treated with heparin. It was then mixed with RPMI-1640 culture medium in equal proportions and the resulting mixture was subjected to centrifugation at 800 g for 15 minutes. After the centrifugation, the peripheral blood mononuclear cells were separated using a Ficoll gradient. The peripheral blood mononuclear cells were then suspended again in RPMI-1640 and placed in T25 flasks. The flasks were incubated at 37 °C for 2 hours. After the monocyte adherence to the plate, the supernatant, which contains all mononuclear cells except monocytes, was removed by gently washing twice.DMEM medium (with 10% FBS and 50% conditioned media or negative control media) was used to culture of the monocytes. After 24 hours, 5-10 mL of cold PBS buffer without calcium and magnesium with EDTA (10 mM) and 4 mg/mL lidocaine were added to each flask and the flasks were gently shaken for 5-10 minutes until the cells were separated. And the purified cells were examined microscopically.After 20 minutes, 150 μL of DMSO was added and the color intensity was read at 490 nm.

###  MTT assay

 To assess vitality, monocyte suspension was added to a 96-well plate and incubated for 4 hours. 20 µL of MTT solution (5 mg/mL) was added to the wells andthe result was read at a wavelength of 492 nm.

###  Neutral red assay

 In order to evaluate ability to pick up neutral red by monocytes,a solution of 0.33% neutral red (Sigma-USA) was added to the monocyte’s culture medium of %10 w/w and then incubation at 37 ˚C for 2 hours was performed.The culture medium was removed and the cells were washed three times with phosphate buffer.DMEM medium contains 1% acetic acid and 50% ethanol was added and the cells were incubated for 10 minutes with gently shaking.Finally, the optical density of solution was determined with an ELISA reader on wavelength of 540 nm.

###  Phagocytosis assays

 1 × 10^6^ cell/mL monocyte suspension was incubated with 100 µL of mouse serum for 5 minutes.Then, 100 µL of *Candida albicans* yeast (1 × 10^6^ cells/mL) in PBS buffer was added to the tube.At 0 and 90 minutes time point, 10 µL prepared suspension was taken and 10 mL distilled water (pH: 11) was added to it.100 µL of the final suspension was added to the 96-well plate and MTT assay as already described was done. Finally, the result was read at a wavelength of 492 nm. The following equation was used to calculate the killing percentage:


$$KILLING\;\%=100-\frac{100\times OD\;T90}{OD\;T0}$$

In order to determine the phagocytosis percentage, Non-phagocytosed bacteria (E) using subtractive centrifugation (150 g at 4 °C for 10 minutes) were isolated after 90 min of incubation. MTT assay as already described was done. Finally, the result was read at a wavelength of 492 nm.


$$PHAGOCYTOSIS\;\%=100\times \left(OD\;T90-\frac{\frac{E}{G}}{OD\;T0}\right)\;G=\frac{OD\;C90}{OD\;C0}$$

###  Phenotypic characterization of MSCs and monocytes

 The surface marker expression of MSCs and M2 cells was analyzed using flow cytometry. For identifying MSCs, the monoclonal antibodies including anti-CD90, anti-CD29 and anti-CD45 and for identifying M2 cells, the monoclonal antibodies including anti-CD68, and anti-CD206 were used. As previously described, 1 × 10^6^ cells from each cell were stained with the specific antibodies and incubated at room temperature for 1 hour in darkness. The cells were washed with PBS and then analyzed with FACSCalibur flow cytometry. Flow Jo software (version 7.6) was used for analyzing data.^21,22^

###  Co-culture of MSCs and macrophages 

 In this experiment, we added 100 µL of MSC suspension (with a cell concentration of 2 × 105 cells/mL) to individual wells of 96-well microplates that already contained macrophages. The plates were then incubated for duration of 4 hours at 37 °C in a humidified atmosphere with 5% CO2.

###  Cytokine assay (function validation)

 For this purpose, the macrophages cells were stimulated with tetradecanoyl phorbol acetate (100 ng/mL) and the production of IL-10, IL-4, TGF-β and IL-12 cytokines in their supernatant was measured.To measure cytokines, ELISA kits (Peprotech, Iran) were used according to the manufacturer’s instructions and its results were read by ELISA reader (Novin Gostar Company, model DANA3200, Iran). The measurement of each of the cytokines IL-4, INF-γ, IL-17, IL-10 and TGF-β was done separately and by the sandwich ELISA method as follows. Briefly, the desired antibody was diluted with PBS (1 μg/mL) and 100 µL of diluted antibody was added to the wells and incubated overnight. After washing, 300 µL blocking buffer was added to the wells and incubated for 1 hour.100 µL of the macrophagessupernatant/standard samples was added to the wells and incubated for 1 hour. In the next step, the biotinylated antibody (0.5 μg/mL) was added and incubated for 2 hours. Then Streptavidin-HRP conjugate was diluted 1 to 20 000 and 100 µL was added to each well and incubated for 30 minutes. The substrate solution was added to each well and incubated for 20 minutes.HCL 1M was used to stop the reaction and finally OD was read by ELISA reader at 450 nm. All of incubations were done at room temperature and between all of steps; washing was performed 4 times with PBS.

###  Animals

 All animal experiments were performed according to the guidelines approved by University’s ethics committee (Ethic NO. IR.BMSU.REC.1398.301). The female C57BL/6 mice with an age range of 6 to 8 weeks (purchased from Pasteur Institute of Iran) were applied for this research. Animals were kept in standard conditions (temperature of 18–22 °C, 12 h light and 12 h darkness, and 55 ± 5% humidity).

###  Induction of arthritis 

 At first the emulsion of collagen type II and Freund’s Complete Adjuvant (FCA) was prepared as follows: Collagen II from the bovine nasal septum (Sigma-Aldrich Co. LLC., cat. No. C7806-10 MG) was mildly dissolved in a dilute solution of 0.05 M acetic acid at the final concentration of 4 mg/mL by stirring overnight at 4 °C and then was emulsified with FCA in a one-to-one ratio in an ice-water container using a high-speed homogenizer (1000 rpm, 60 min).^23,24^ Afterwards, to create CIA ^13^ in mice, 0.1 mL Collagen II - FCA emulsion was injected subcutaneously into the root of the tail and 0.1 mL (as the second dose) was injected subcutaneously into the left hind footpad (the starting point of inflammation) with a short interval after the first dose.^9,25^

 After a week, the booster injection was done with the same two doses.

 Maximum inflammation was observed on the 14th day after the booster injection (the 21st day after the first injection), and then, the treatment was started and lasted for 4 weeks.

###  Treatment

 The 45 female mice were randomly divided into the five groups as following: (1( M2-mediated cell therapy group; induced with RA and receiving anti-inflammatory M2 (2 × 10^6^ cells) every two weeks by two intraperitoneal and intravenous injections (for systemic effectiveness) and also one injection in the synovial fluid of the foot joint (for local effectiveness), (2) The group treated with IL-1RA; induced with RA and receiving mouse recombinant IL-1RA (3 mg/kg) every day by subcutaneous injections in two areas, the back of the neck (systemic effect) and the toes (local effect), (3( The combinatory therapy group treated with M2 and IL-1RA; induced with RA and treated with anti-inflammatory M2 (2 × 10^6^ cells) along with mouse recombinant IL-1RA as explained earlier, (4( patient control group; induced with RA and not receiving any treatment, (5) healthy control group; not induced with RA and not receiving any treatment.

###  Symptoms evaluation of arthritis

 The arthritis improvement was assessed using three indicators.^24,26^ To measure paw volume, a weighing method was employed, wherein the paw was submerged in water until reaching a designated point on the scale. The average volume was then calculated based on the water density at 25 °C, approximately 0.9970 g/mL, which is close to 1 (weight equals volume).^27^ The severity of CIA^13^ in the paws was evaluated using a scoring system as follows: 0 represented normal, 1 indicated mild swelling and redness, 2 denoted moderate swelling and redness, 3 signified severe swelling and redness in the limb, and 4 indicated pronounced swelling, redness, and inability to use the limb.^18^ The percentage of anti-inflammatory activity on days 14 and 21 was determined using the following formula^24^: anti-inflammatory activity = [1 − Vt/Vb] × 100, where Vt represented the paw volume in the treatment groups and Vb was the paw volume in the control group.

###  Spleen isolation; evaluation of spleen weight index and lymphocyte proliferation index

 After the completion of the treatment period, following the blood collection and killing of the mice, their spleens were separated and weighed under the sterile conditions. First, the spleens were fragmented and then crushed into 5 mL RPMI-1640 supplemented with %10 FBS. The cell suspension was centrifuged at 200 g for 10 minutes. Then, cell pellet was suspended with 5 mL lysate buffer to remove its RBCs. After centrifugation and resuspension, 1 × 10^5^ cells were added to each well of 96 well plate. Collagen (1 mg/mL) as antigen was added to some well and the plate was incubated at 37 °C for 72 hours. Some wells were considered as without antigen and some of them as blank. MTT solution was added to the wells and the plate incubated at 37 °C for 4 hours. after that, DMSO was added to the plate and the color intensity was read at 490 nm and the stimulation index was calculated as follow; Stimulation index = OD with collagen-OD blank/ OD without collagen-OD blank.

###  C-reactive protein (CRP) and rheumatoid factor (RF) measurement 

 After serum deactivation (30 minutes at 56 °C), a drop of serum was placed on a slide.Then a drop of antibody solution bound to the latex was added.The slide was rotated with the circular movement of the hand for 3 minutes and the agglutination intensity was reported.

###  Evaluation of nitric oxide and myeloperoxidase (MPO) 

 The amount of NO production was determined by grease colorimetric method,^18^ as previously explained. For MPO analysis, 10 µL sample was mixed with 80 µL H2O2 (0.75 mM) and 110 µL TMB solution in a 96-well plate. The plate was incubated at 37 °C for 5 minutes and after adding stop solution, the color intensity was read at 490 nm by ELISA reader.

###  Cytokine assay

 The spleen cells were cultured in a T25 culture flask with 5 mL RPMI-1640 supplemented with 10% FBS and 100 U/mL penicillin–streptomycin and incubated at 37 °C and 5% CO2. After 72 hours, the supernatant was isolated to evaluation of TNF-α, INF-γ, IL-17, IL-10 and TGF-β cytokines. The measurement of each of the cytokines IL-4, INF-γ, IL-17, IL-10 and TGF-β was done separately and by the sandwich ELISA method as previously described.

###  Histopathological assessments 

 After euthanize the mice, their hind legs were placed in 10% formalin solution for several days so that their tissue is completely fixed. After decalcification of the bone, the tissue was embedded in paraffin, and cross-sections were prepared and mounted on slides for histopathological evaluation. The tissue sections of the joints were stained with hematoxylin-eosin (H & E) and the degree of inflammation and changes in the joints were evaluated by microscopy (Olympus BX43, Shinjuku, Japan). Scoring was done from 0 to 5; Zero score: indicates the healthy tissue of the mouse ankle joint (absence of inflammation), Score 1: severe destruction of cartilage that reaches the bone level or hypertrophy of cartilage (weak inflammation), Score 2: indicates the disruption of the cartilage structure (moderate inflammation), Score 3: indicating a decrease in joint space (moderate to severe inflammation), Score 4: indicates severe joint inflammation, Score 5: indicating the formation of pannus, the presence of connective tissue and infiltration of edematous cells. The samples were studied by two researchers in a double-blind manner.

###  Statistical analysis

 One way ANOVA and Tukey’s test were used to compare the parametric data and Kruskal Wallis test was used to compare related non-parametric data by SPSS software (version 21). Data were presented as mean ± standard deviation. *P* < 0.05 was used as the significant level of data.

## Results

###  Recombinant IL-1RA gene designing, expression, purification and validation

**Figure 1:**
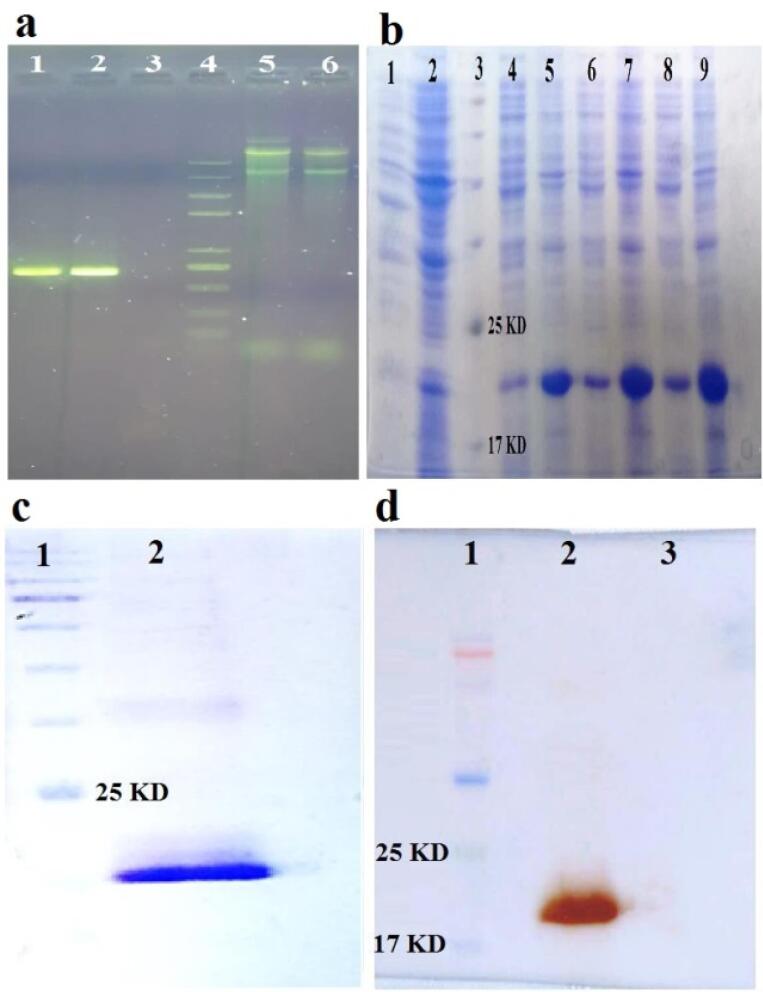
a) Validation of the recombinant construct containing IL-1RA gene in pET-28a^( + )^ vector by PCR on 2% agarose gel. Lane 1 and 2. The expected fragment obtained from the PCR of the recombinant construct containing the IL-1RA gene (~700 bp) in two different colonies, lane 3. PCR negative control, lane 4. DNA size marker, lane 5 and 6. Extracted plasmids from two different colonies. b) SDS-PAGE of the expressed protein and checking its solubility under optimal conditions. 1. Negative control soup (not induced), 2: Negative control pellet (not induced), 3: Protein marker, 4, 6 and 8. Soup of 3 different induced colonies, 5, 7, and 9. The pellets of induced 3 different induced colonies. c) Purified IL-1RA recombinant protein with Ni-NTA column and concentrated with PEG, on SDS-PAGE gel. 1. Protein marker, 2. Final purified and concentrated protein. d) Western blot of the expressed recombinant IL-1RA protein with a specific monoclonal antibody against N-terminal hexahistidine (His_6_-tag). 1. Protein marker, 2. Reaction between the above antibody and the target protein (19.8 kDa), which indicates the correctness of the expressed recombinant IL-1RA protein, 3. Lack of reaction between the above antibody and BSA protein (as negative control)

 The PCR products were electrophoresed on 2% agarose gel and its results showed a band about 700 bp which is related to the *IL-1RA* gene in the investigated colonies (Figure 1a). The confirmation of the recombinant construct containing the *IL-1RA* gene in the pET-28a^( + )^ vector was done by determination of the sequence and the result of the data analysis indicated the correctness of the desired gene sequence and the absence of any changes in the above sequence compared to the optimized sequence (data not shown).

###  Expression, purification and validation of IL-1RA

 After optimization, the best condition for expression included 0.5 mM IPTG at 37 °C for 6 h was obtained. The SDS-PAGE pattern (Figure 1b) showed that the *IL-1RA* gene was expressed in the presence of IPTG as an inducer. The IL-1RA protein had a considerable band at approximately 19.8 kDa. The purification results showed that the recombinant protein was purified properly and a little bit undesired band was observed in the SDS-PAGE (Figure 1c). Western blot analysis verified the purified IL-1RA recombinant protein by anti His_6_-Tag antibody (Figure 1d).

###  Immunophenotyping of mesenchymal stem cells

**Figure 2:**
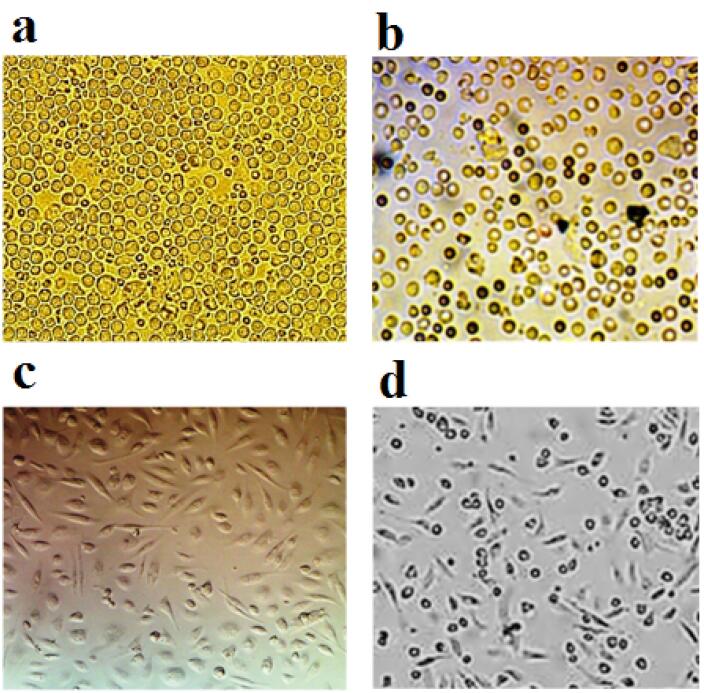
The passage of MSCs aspirated from the bone marrow, so that the change in the shape of these cells after the passage is clearly visible.a) Cells on the second day of cultivation, b) Freshly aspirated cells (first day of cultivation), c) Cells after passage, d) Cells on the fifth day of cultivation

**Figure 3:**
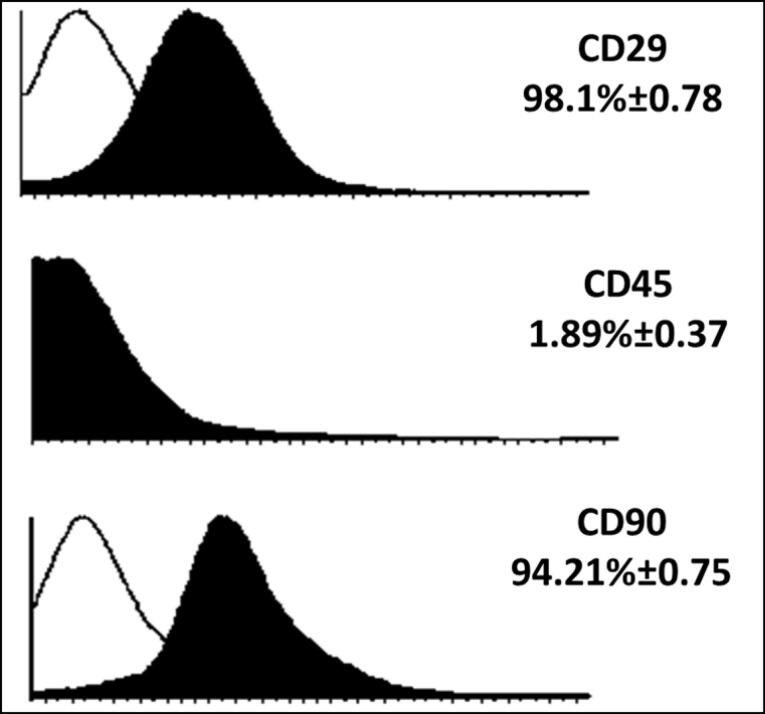
The results of expression analysis of surface markers in mesenchymal stem cells. About 98% of cells had CD29 marker, about 1.9% of cells had CD45 marker and about 94% of cells had CD90 marker

 MSCs extracted from the bone marrow of mice were cultured (Figure 2) and examined for the expression of surface markers CD90, CD29 and CD45. The results showed that the cells used in this study had a high level of CD29 and CD90 (the main MSC markers in mice). While these cells showed a low level of CD45 as the main marker of leukocyte cells (Figure 3).

###  Functional evaluation of isolated monocytes

 The percentage of viable cells, the ability to uptake neutral red and the amount of MTT regeneration were shown in Table 1. After monocyte treatment with opsonized yeast, the phagocytosis, opsonized yeast killing, Nitroblue tetrazolium test(NBT) and NO analysis were performed and also shown in Table 1.

**Table 1:** Results of the physiological functions of isolated monocytes

**Isolation method**	**Lidocaine/ EDTA**
Viable cells (%)	85.3 ± 12.8
Neutral red (OD_540 nm_)	0.063 ± 0.919
MTT (OD_492nm_)	0.033 ± 50.27
Phagocytosis (%)	61.66 ± 3.05
Opsonized yeast killing (%)	50.25 ± 2.08
NBT (OD_492 nm_)	0.659 ± 0.013
NO (%)	71.33 ± 7.09

###  Immunophenotyping, produced cytokines, NO production and respiratory burst of educated and non-educated monocytes

 The monocytes educated and non-educated with the supernatant of MSCs were studied in order to investigate the expression of anti-inflammatory phenotype, i.e. CD68 and CD206 markers.The results showed that the expression of the mentioned markers in the educated monocytes (47.2%) was significantly (*P* < 0.001) higher than non-educated monocytes (24.1%) (data not shown).As we previously verified,^28^ the results of measuring cytokines produced by the monocytes educated with the supernatant of MSCs as well as non-educated monocytes stimulated with 12-O-Tetradecanoylphorbol-13-acetate (100 ng/mL) showed that the production rate of IL-10, IL-4, TGF-β and IL-12 cytokines were different in their supernatant.The production of IL-4 and IL-12 cytokines (indicating the humoral and cellular activity, respectively) was significantly decreased and the production of TGF-β and IL-10 (indicating the anti-inflammatory activity) cytokines was significantly increased in educated monocytes compared to non-educated monocytes (*P* < 0.05).The results of NBT reduction and nitrate reduction in Gries’s test showed that the non-educated monocytes in causing inflammation was significantly stronger than educated monocytes (*P* < 0.001) (Table 1).

###  Animal findings

####  Qualitative and quantitative assessments of inflammation

**Figure 4:**
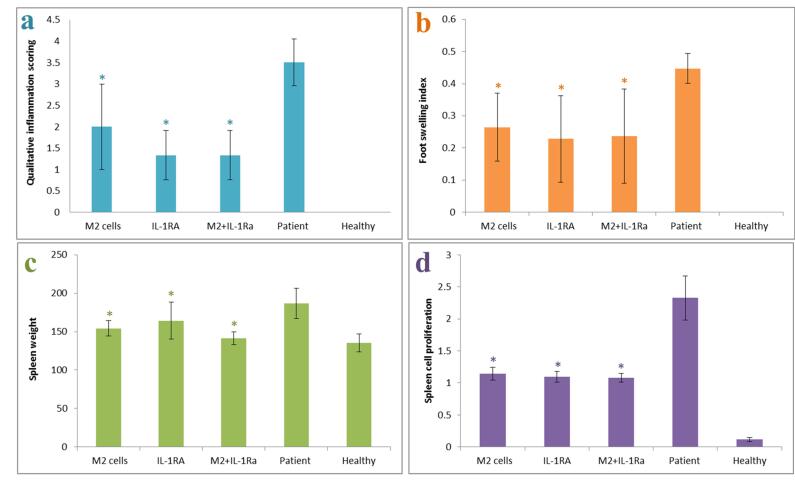
Inflammatory symptoms scoring in mice with RA, after treatment with M2 cells, IL-1RA and also combinatory method (M2 cells + IL-1RA) compared with patient and healthy control groups. a. Inflammatory symptoms scoring, b. Foot swelling index, c. Spleen weight and d. Proliferation of spleen cells. *Value significantly different from controls (*P* < 0.05). **Value significantly different from other groups with different treatments (*P* < 0.05)

 The results showed that the patient control group had the highest inflammation score (most inflammatory symptoms) and the highest swelling rate, the healthy control group had zero score (no symptoms) and no swelling and the group treated with IL-1RA and combinatory therapy group had the lowest inflammation score (least inflammatory symptoms) and minimal leg swelling. The results of spleen weight showed that the patient control group had the highest spleen weight, and the healthy control and combinatory therapy groups had the lowest spleen weight. The results showed that the patient control group’s spleen cells had the highest proliferation rate (indicating the severity of destructive immune responses) compared to the healthy control group and the groups treated with IL-1RA, M2 cells and also combinatory therapy group (Figure 4).

###  Qualitative results of serum CRP and RF

**Figure 5:**
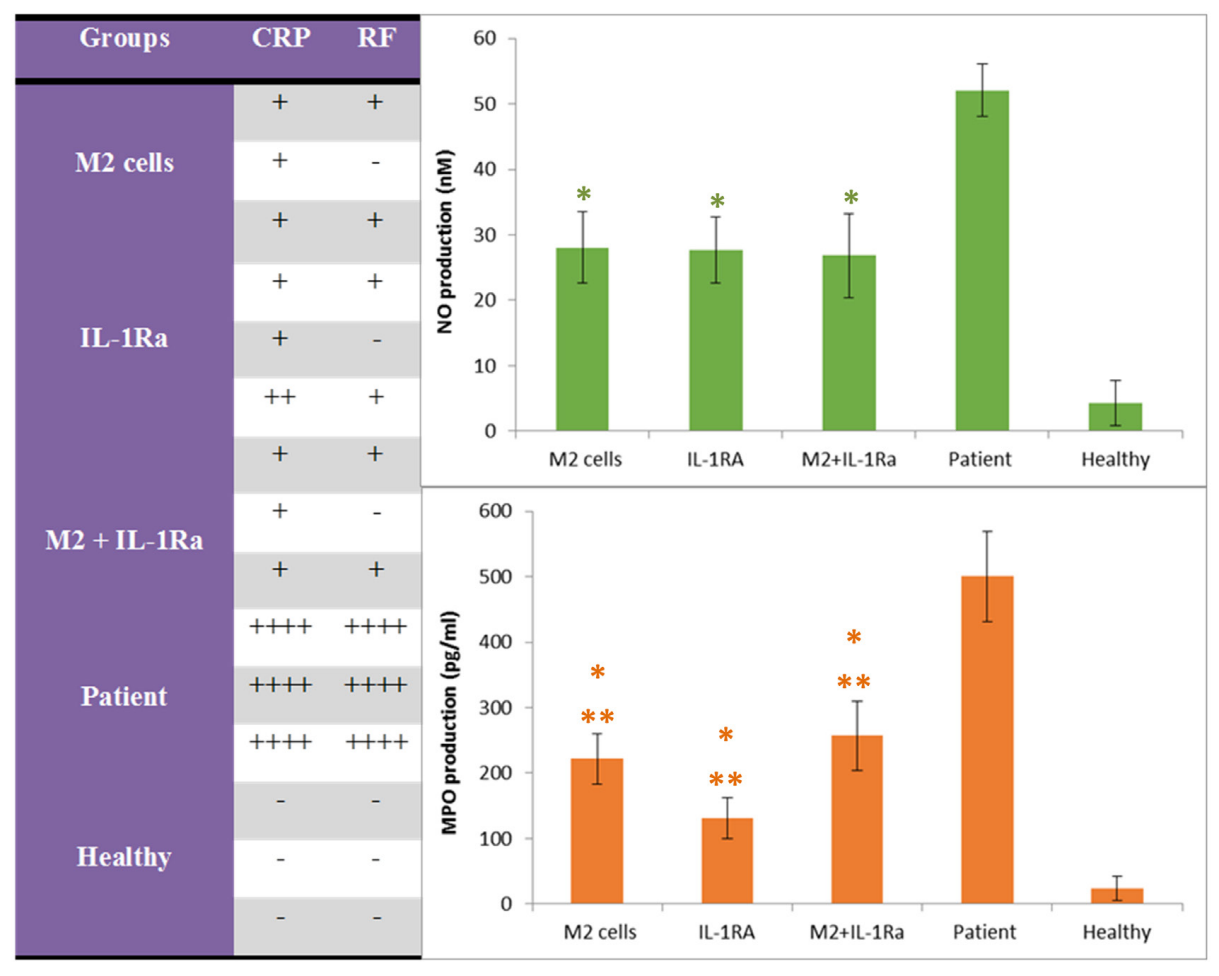
The amountof CRP, RF, NO and MPO production in the serum of mice with RA after treatment with M2 cells, IL-1RA and combinatory method(M2 cells + IL-1RA) compared with patient and healthy control groups. *Value significantly different from controls (*P* < 0.05). **Value significantly different from other groups with different treatments (*P* < 0.05)

 The results of serum CRP showed that the patient control group had the highest CRP level compared with the treated groups. The healthy control group was CRP negative.The results of serum RF showed that the patient control group had the highest amount of RF production compared with the treated groups and the healthy control group was RF negative (Figure 5a).

###  The results of NO and MPO production

 The results of NO showed that the patient control group had the highest amount of NO production, the healthy control group had a negligible amount (close to zero) and the treated groups had a relatively similar situation and the lowest amount of NO production (Figure 5b).

 The results of MPO showed that the patient control group had the highest amount of MPO production, the healthy control group had a negligible amount (close to zero) and the group treated with IL-1RA had the lowest amount of MPO production. Although the groups those treated with educated monocytes (M2) and combinatory therapy also showed a favorable condition in comparison (Figure 5c).

###  Measurement of secreted cytokines

**Figure 6:**
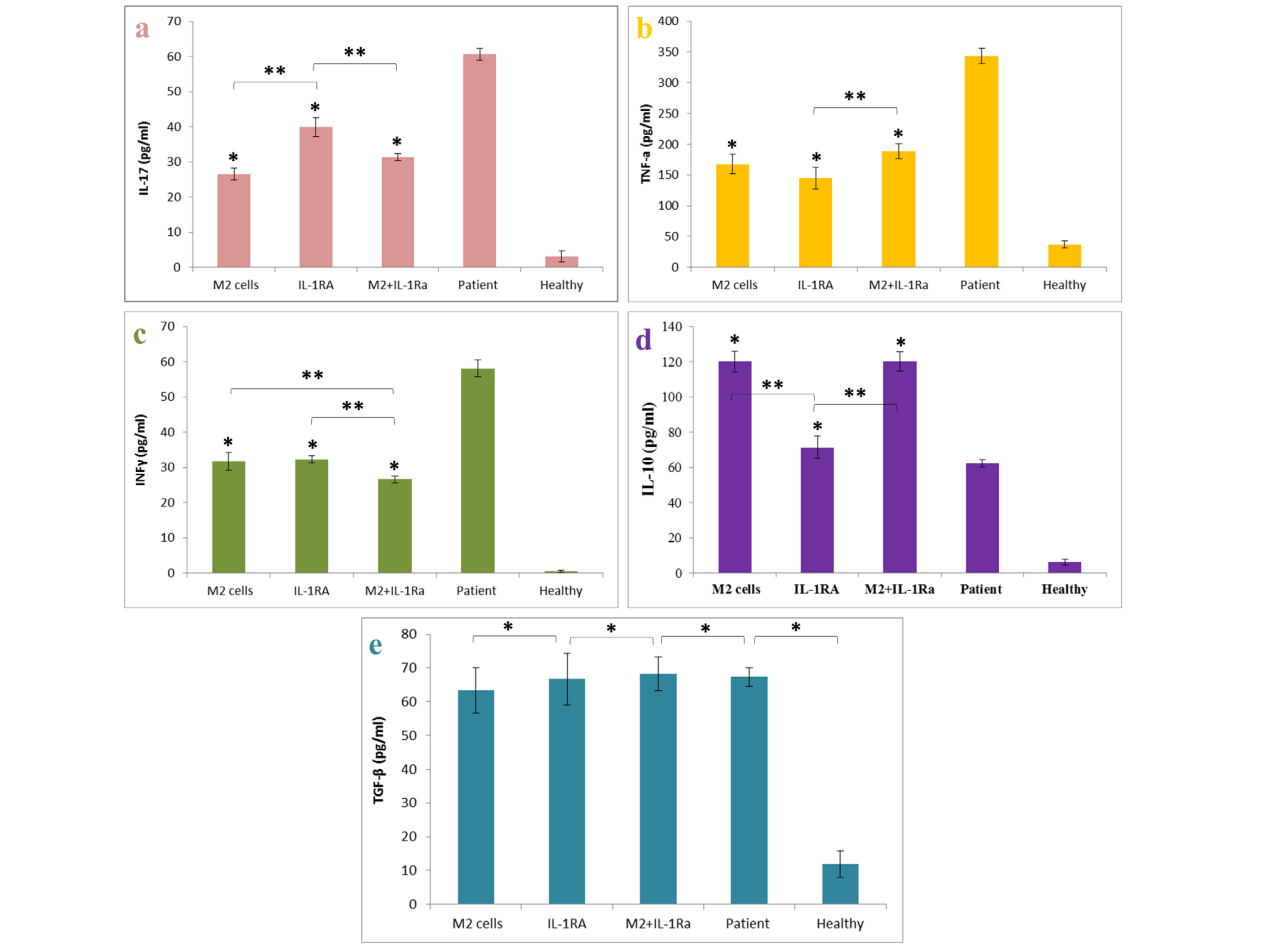
The amount of IL-17, TNF-α, INFγ, IL-10 and TGF-β in the spleen supernatant of mice with rheumatoid arthritis after treatment with M2 cells, IL-1RA, combination treatment (M2 cells + IL-1RA) compared with patient and healthy control group. a. The IL-17 cytokine production after treatment with noted groups, b. The TNF-α cytokine production after treatment with noted groups, c. The INFγ cytokine production after treatment with noted groups, d. The IL-10 cytokine production after treatment with noted groups and e. The TGF-β cytokine production after treatment with noted groups. *Value significantly different from controls (*P* < 0.05). **Value significantly different from other groups with different treatments (*P* < 0.05)

 The results of examining the amount of cytokines produced and secreted by spleen cells showed that the IL-17, TNF-α and also INF-γ level decreased significantly with the improvement of the severity of the disease in the groups treated with educated monocytes (M2) and also the IL-1RA. Combinatory therapy also indicated the existence of a synergistic effect in reducing the INF-γ (as the Th1 and cellular immunity index) level. Also the results showed that the IL-10 level increased significantly with the improvement of the severity of the disease in the group treated with educated monocytes (M2) and combinatory therapy group without significant decrease in TGF-β level compared with patient control group (Figure 6).

###  Findings of histopathological studies

**Figure 7:**
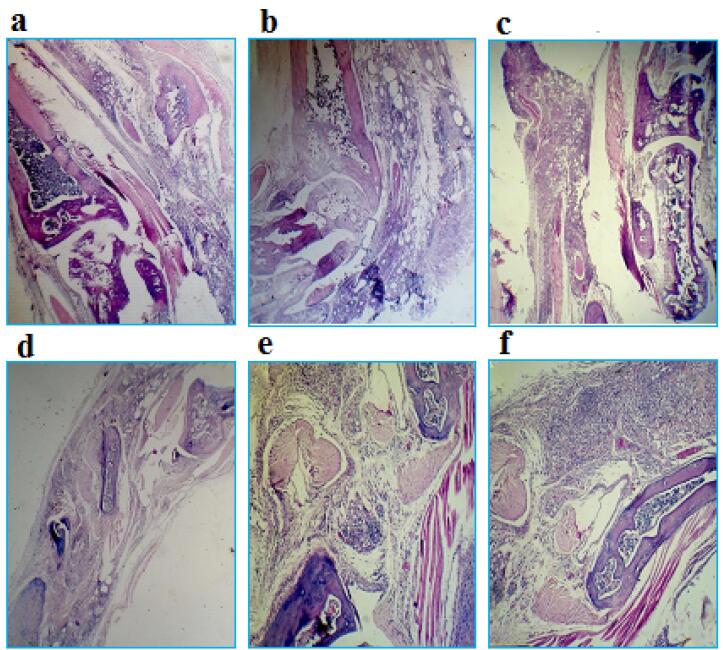
Sections used to foot joint tissue damage scoring in a mouse model of RA with 40x magnification. a. Zero score: indicates the healthy tissue of the mouse ankle joint (absence of inflammation), b. Score 1: severe destruction of cartilage that reaches the bone surface or cartilage hypertrophy (weak inflammation), c. Score 2: indicates the disorder of cartilage structure (moderate inflammation), d. Score 3: indicating a decrease in joint space (moderate to severe inflammation), e. Score 4: indicates severe joint inflammation and f. Score 5: indicating the formation of pannus, the presence of connective tissue and infiltration of edematous cells

**Figure 8:**
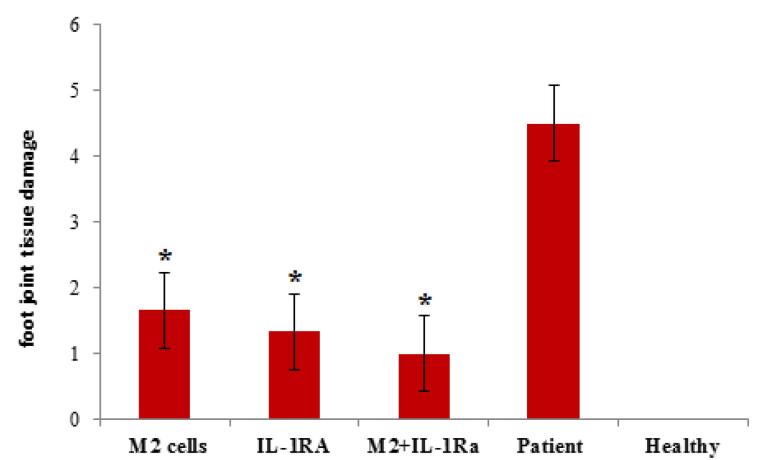
The diagram related to the histopathology results of the groups of mice suffering from rheumatoid arthritis, after the treatment procedures. The scoring results related to the groups treated with IL-1RA, with cell therapy by M2 cells and also with the combinatory therapy resulting from the synergistic effect of the two treatments. *Value significantly different from controls (*P* < 0.05). **Value significantly different from other groups with different treatments (*P*< 0.05)

 In Figure 7, sections used for scoring foot joint tissue damage in a mouse model of RA are shown. These tissue sections were stained with hematoxylin-eosin and observed with a light microscope with 40x magnification.The results of the microscopic examination of tissue pathology slides showed that the patient control group (with an average score of 4.5) had the most tissue damage, while the healthy control group (with an average score of zero) had no damage. The groups treated with IL-1RA (average score 1.33) and also with combinatory therapy (average score 1) also had the least tissue damage.In fact, the results of this part indicated the efficiency of the two treatment methods proposed in this research, compared to the other mentioned treatment methods. It also showed that the combinatory therapy had a synergistic effect and the least tissue damage among the treated patient groups (Figure 8).

## Discussion

 Monocytes, macrophages and dendritic cells are very important in broad range of immunology research, especially in studies related to immunotherapy for cancer and autoimmune disorders.^29-31^ When inflammation happens, monocyte/macrophage cells gather to the injury place. New researches show that especially alveolar macrophages change in different conditions. The existence of different types of macrophages shows that subject is important.^32,33^ Alternative and M2 monocytes/macrophages produce less inflammatory agents. They work like regulators and suppress inflammation by producing regulatory factors and increasing the process of removal of useless and damaged cell components (phagocytosis).^33-35^ Previously, scientists have shown that MSCs and regulatory T cells by producing IL-10 and indoleamine 2 and 3 dioxygenase lead to the formation of type of monocytes/macrophages called M2c macrophages.^36^ Many studies confirmed it. IV injection of 10^6^ cells of two subtypes namely: M2a (produced by IL-4 and IL-13), and M2c (produced by IL-10 and TGF-β) can suppress inflammation. But it should be noted that M2c cells is more effective in reducing tissue damage and fibrosis than M2a macrophages.^37^ The results of our study showed that two intravenous injections of 10^6^ monocyte/macrophage cells treated with the supernatant derived from MSCs significantly reduces inflammation induced by type 2 collagen. It should be noted that the cost of producing macrophages in this study is more suitable compared to cell polarization with the help of cytokines.

 Our Flow cytometry analysis showed that the percentage of CD68 + /CD206 + monocytes is higher in the group that was treated with MSC supernatant compared to the untreated group.Examining the simultaneous expression of CD68 and CD206 markers is a well-known method for determining type 2 macrophages using flow cytometry.^38^ CD68 is a glycoprotein capable of binding to low-density lipoproteins, which is expressed on monocyte/macrophage cells.^39^ CD206 (mannose receptor) plays an important role in increasing the phagocytosis and clearance ability of M2 macrophages compared to M1 macrophages.^33,40^ An increase in the production of reactive oxygen and nitrogen species indicates an increase in the inflammatory activity of the target cell.^33^ The results of our study showed that the supernatant derived from mouse MSCs suppresses the production of NO in the educated monocytes.

 Earlier investigations have documented that M2 macrophages, particularly M2c macrophages, release substantial quantities of IL-10 and TGF-β to stimulate regulatory T cells and terminate inflammation induced by Th2 cells.^36,37^ Our study findings revealed that educated monocytes demonstrate a notable increase in the production of anti-inflammatory cytokines IL-10 and TGF-β, as opposed to non-educated monocytes.

 Regarding IL-10 and TGF-β, these two cytokines together have a modulating effect on the immune system. IL-10 and TGF-β can’t perform this role alone so that if only one of them comes up, maybe not desirable and can even increase the severity of the inflammatory disease. Therefore, the amount of these cytokines in the treated groups as well as the patient control group had a much higher level than the healthy control group, which can indicate the reactions of the immune system against collagen II in CIA pathogenesis and also the natural reaction of immune system for reducing inflammatory responses in the treated groups. While in the patient control group, the level of TGF-β was relatively high (closeto the treated groups), but the level of IL-10 was average (between the upper and lower limits). In the treated groups, there was an increase in the IL-10 compared to the patient control group. The highest increase of IL-10 level occurred in the group treated with educated monocytes (M2) and also combinatory therapy. These groups showed the best condition after the healthy control group among the others which it is maybe due to the high level of both IL-10 cytokines and TGF-β. This indicates the efficiency of cell therapy with educated monocytes (M2) and also combinatory therapy proposed in this research.

 A very important point is that TGF-β with IL-17 and IL-6 has an inflammatory effect, but the TGF-β cytokine combined with high levels of IL-10, shows an anti-inflammatory and a modulating effect on the immune system. Therefore, in the patient control group, where the level of IL-17 was also very high, the high amount of TGF-β with an average level of IL-10, causedincreased inflammation.

## Conclusion

 In conclusion, this research contributes to the advancement of our understanding of the role of MSCs and M2 macrophages in treating inflammatory disorders, specifically RA. The proposed approach holds promise for future therapeutic applications, potentially offering an alternative or complementary treatment option to current strategies. It is recommended that future research focuses on refining the production of M2 macrophages and conducting comprehensive clinical trials to assess their effectiveness in real-world settings.Overall, by elucidating the potential of MSC-induced M2 macrophages in reducing inflammation and highlighting the synergistic effects of combinatory therapy, this study paves the way for further investigations in this field. The findings have significant implications for the development of novel treatment approaches for inflammatory diseases and provide valuable insights for researchers and clinicians alike.

## Acknowledgments

 The authors would like to thank Dr. Abdullah Moridikia, the researcher of Baqiyatallah University of Medical Sciences for assisting in the animal phase of this study.

## Competing Interests

 The authors have no relevant financial or non-financial interests to disclose.

## Consent to Participate

 Not applicable.

## Data Availability Statement

 All data analyzed during this study are included in this article.

## Ethical Approval

 All assessments were conducted in accordance with ethical principles and under the supervision of the University’s Ethics Committee (Ethic NO. IR.BMSU.REC.1398.301).
